# Molecular characterization of orf virus from sheep and goats in Ethiopia, 2008–2013

**DOI:** 10.1186/s12985-016-0489-3

**Published:** 2016-02-29

**Authors:** Esayas Gelaye, Jenna Elizabeth Achenbach, Shiferaw Jenberie, Gelagay Ayelet, Alebachew Belay, Martha Yami, Angelika Loitsch, Reingard Grabherr, Adama Diallo, Charles Euloge Lamien

**Affiliations:** Animal Production and Health Laboratory, Joint FAO/IAEA Division of Nuclear Techniques in Food and Agriculture, Department of Nuclear Sciences and Applications, International Atomic Energy Agency, Wagramer Strasse 5, P.O. Box 100, A-1400 Vienna, Austria; Institute of Applied Genetics and Cell Biology, University of Natural Resources and Life Sciences, Muthgasse 18, A-1190 Vienna, Austria; Research and Diagnostic Laboratories, National Veterinary Institute, P.O. Box 19, Debre Zeit, Ethiopia; Institute for Veterinary Disease Control, Austrian Agency for Health and Food Safety, Robert Koch-Gasse 17, A-2340 Mödling, Austria; Institute of Applied Microbiology, University of Natural Resources and Life Sciences, Muthgasse 11, 1190 Vienna, Austria

**Keywords:** A32L gene, B2L gene, Ethiopia, Goat, Orf virus, Sheep

## Abstract

**Background:**

Orf is a contagious disease of sheep, goats and wild ungulates caused by orf virus (ORFV) a member of the genus Parapoxvirus, Poxviridae family. Although orf is endemic in Ethiopia, little attention has been given so far as it is not a notifiable disease by the World Organization for Animal Health. In this work, we have investigated orf outbreaks representing five different geographical locations of Ethiopia, in Amba Giorgis, Gondar zuria, Adet, Debre zeit and Adami Tulu, between 2008 and 2013.

**Results:**

The viral isolation and the sequence analysis of the A32L and the B2L genes of eighteen representative isolates confirmed that sampled animals were infected by ORFVs.

The phylogenetic study and the comparative analysis of the deduced amino acid profile suggests that there were two main clusters of ORFV isolates which were responsible for the investigated outbreaks. Additionally the analysis of these two genes showed limited variability to ORFVs encountered elsewhere. This is the first report on the genetic characterization of the ORFV isolates from sheep and goats in Ethiopia.

**Conclusion:**

The molecular characterization of Ethiopian ORFV isolates highlighted the circulation of two main clusters causing orf disease in sheep and goats. The use of laboratory based methods and a constant monitoring of Ethiopian ORFV isolates is needed to better understand the dynamic of ORFV circulating in the country and facilitate the implementation of control measures.

**Electronic supplementary material:**

The online version of this article (doi:10.1186/s12985-016-0489-3) contains supplementary material, which is available to authorized users.

## Background

Orf is an acute, contagious, debilitating and economically important zoonotic viral skin disease of sheep, goats and wild ruminants caused by orf virus (ORFV). ORFV, a prototype of *Parapoxvirus* genus of *Chordopoxvirinae* subfamily within the *Poxviridae* family, has a size of approximately 260 nm length and 160 nm width comprised of a linear double-stranded DNA genome (134–139 kbp) with high GC content (63–64 %) in comparison with other poxviruses [1–5]. The genus *Parapoxvirus* also includes pseudocowpox virus (PCPV), bovine papular stomatitis virus (BPSV) and parapoxvirus of red deer in New Zealand [3–5].

ORFV infection in sheep and goats is generally known as orf, contagious ecthyma, infectious labial dermatitis, scabby mouth, contagious pustular dermatitis, or sore mouth. Lesions of orf progress from erythema, vesicle formation, pustules and then scabs [3–6]. The disease not only has an economic impact on farmers worldwide but also has a considerable negative effect on animal welfare. Infected animals are sickly, fail to thrive, and are more susceptible to bacterial infections. Characteristic of the disease are proliferative and often self-limiting lesions (3–4 weeks) on the skin of the lips, on the oral mucosa and around the nostrils. Lesions can also be found occasionally on the teats of nursing animals but rarely on other organs [3–6]. Depending on the location of the lesions, animals may be unwilling to nurse, eat, or walk. The mortality rate related to orf is usually low, but it may be very high when bacterial or fungal secondary infections occur.

The disease also has zoonotic potential, although it is more of occupational hazard to people working with animals (farmers, animal caretakers, veterinarians), characterized by nodular and papillomatous lesions mainly on the hands, face, and mouth [3, 5–7].

A number of natural outbreaks of suspected infections of ORFV are frequently observed in sheep and goats reared under different production systems in Ethiopia. Until now only the observation of clinical signs formed the basis of orf diagnosis in the country. Owing to the existence of several diseases which can potentially present similar lesions on the mouth and related symptoms such as sheep pox, goat pox, peste des petits ruminants, dermatophylosis and foot and mouth disease, laboratory diagnostic of orf is urgently needed in the country to confirm any suspected orf outbreaks. Even though outbreaks of ORFV infections are endemic and develop severe ecthyma lesions in sheep and goats in most of the agro-climatic regions of Ethiopia, no attempt has been made until now to isolate, identify and molecularly characterize the circulating virus isolates. Orf disease is neglected by the veterinary service, however, owing to its zoonotic potential and impact on animal production, more attention needs to be given to the disease.

Two genes are widely used to molecularly characterize ORFV and reveal the genetic variation of parapoxviruses: the A32L gene homologue of vaccinia virus, encoding the viral ATPase protein which plays a role in viral DNA packaging [1, 4, 8–11], and the B2L gene encoding the major virus envelope protein [1, 4, 8–11]. Five functional predicted motifs (motif I-V), which have synergistic activities on the virus-host cell interaction through enzymatic processes, characterize the ATPase protein sequence [2, 4, 12–15]. The C-terminal of the ATPase gene displays high heterogeneity, which makes it suitable for viral strain differentiation [2, 12].

In the present study, we have investigated suspected orf outbreaks occurred at different geographical locations of Ethiopia between 2008 and 2013, and have confirmed the disease and characterized the isolates. The analysis of the isolates, their comparison among each other and to foreign isolates and the implications are discussed to allow a better understanding of ORFV dynamics in the country.

## Methods

### Outbreak areas

**Fig. 1:**
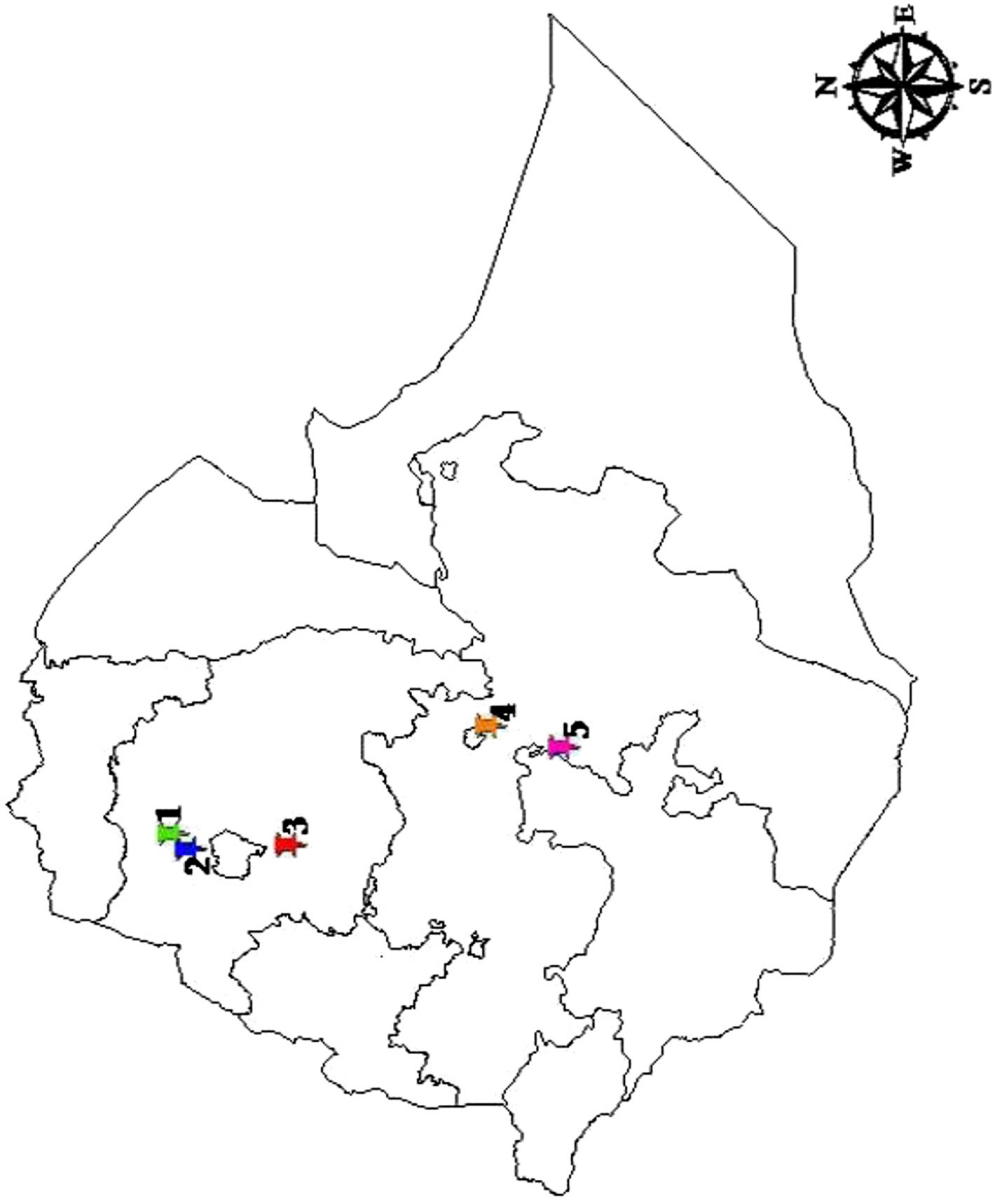
Study area. Representative scabby lesion samples have been collected during orf suspected outbreaks from clinically sick sheep and goats in: 1. Amba Giorgis, 2. Gondar zuria, 3. Adet, 4. Debre zeit, and 5. Adami Tulu. The map was sketched using ArcGIS 9 software (ArcMap™ version 9.3, California, USA)

From 2008 to 2013, pox crusted samples were collected from sick sheep and goats presenting pox-like lesions and suspected to have pox disease. The samples were collected from the five following geographical locations of Ethiopia: Northern Ethiopia (Amba Giorgis [12°46̍05.52̎ N, 37°37̍46.76̎ E], Gondar zuria [12°35̍59.54̎ N, 37°28̍00.36̎ E], and Adet [11°15̍51.36̎ N, 37°29̍31.72̎ E]), central Ethiopia (Debre zeit [8°45̍32.12̎ N, 39°01̍19.87̎ E]), and southern Ethiopia (Adami Tulu [7°51̍33.20̎ N, 38°42̍24.72̎ E]) (Fig. 1). Adet and Adami Tulu are agriculture research centers where animals were kept in a controlled environment while the remaining three outbreaks occurred in herds where animals are reared by individual owners and graze on communal grazing land. All six outbreaks were attended by veterinary professionals.

### Sample and outbreak data collection

Representative tissue samples of scab lesion scrapings were collected from a total of 24 sheep and goats showing suspected clinical signs of poxvirus infection. About 2 g of tissue samples were collected from each animal and placed in a labeled sterile universal tube containing phosphate buffered saline (PBS), pH 7.2 supplemented with antibiotics and antifungal. Samples were immediately transferred into a cold box and transported to the National Veterinary Institute using the cold-chain system. The samples were then stored at −20 °C until laboratory analysis. All the clinical parameters were recorded during collection of samples.

For all outbreak areas, a physical inspection of the diseased sheep and goats was undertaken by veterinary professionals to record the clinical observations. Additional data were collected through an interview of the farmers and animal keepers using a questionnaire (Additional file 1: Table S1). These data included: vaccination history, mortality and morbidity rates, and date of appearance of the disease in the farm or herd, as well as the appearance of lesions on humans in close contact with the diseased animals.

### Ethics statement

Samples were collected on diseased animal during outbreak for disease confirmation. No animal experiment was conducted. Sample collection and their use were approved by the joint animal ethical committee of the National Veterinary Institute and the College of Veterinary Medicine and Agriculture, Addis Ababa University. All efforts were made to minimize animal suffering during the course of sample collection in orf suspected outbreak areas. Consent was obtained from the animal owners for the collection of tissue samples by a veterinarian.

### Virus isolation

Samples were washed three times with sterile PBS containing antibiotics and antifungal. Tissue homogenates (10 % w/v) were prepared using sterile PBS and centrifuged at high speed using a refrigerated centrifuge. 0.5 mL of the supernatant was inoculated onto a confluent layer of Vero cells grown in a 25 cm^2^ tissue culture flask containing10mL Glasgow Minimum Essential Medium (Sigma-Aldrich) supplemented with 10 % fetal calf serum (Gibco). The inoculated cultures were incubated at 37 °C, 5 % CO_2_ and observed daily for the appearance of virus-induced cytopathic effects (CPE). Samples were considered negative when no CPE was observed following three blind passages.

### Virus DNA extraction and preliminary screening by PCR

The pathological tissue homogenates (10 % w/v in PBS) were centrifuged at 10,000 g for 10 min at +4 °C. The supernatant was used for virus DNA extraction using the DNeasy Blood and Tissue kit (Qiagen) following the manufacturer’s instruction. The eluted DNA was labeled and stored at −20 °C until further testing. All samples were first screened by ORFV specific PCR [16], using a C1000 Touch thermal cycler (Bio-Rad, Foster City, CA, USA).

### Amplification, cloning and sequencing of fragments containing the full A32L and B2L genes

Primers (Table 1) were designed for the amplification of fragments containing the full B2L and A32L genes using Allele ID version 6 software package (Premier Biosoft International, Palo Alto, CA, USA), using ORFV strain OV-SA00 as template (Accession number: NC005336); The primers binding sites in the genome for B2L gene were ORFV-B2Lf-For =10838-10857 and ORFV-B2Lf-Rev =12030-12047; and for A32L gene ORFV-A32f-For =110603-110622, and ORFV-A32f-Rev = 111681-111700). Internal primers were also designed for sequencing (Table 1). Primers were synthesized by VBC Biotech, Austria.

**Table 1:** Primers used in this study. Primers orf1 and orf2 [16] were used for the preliminary screening of the clinical samples. The remaining primers were used for the amplification and sequencing of the full-length A32L (825 nt) and B2L (1137 nt) genes of ORFV. The estimated PCR product sizes are presented

SI No	Primer name	Sequences (5'– 3')	Application	PCR product size (base pair)
1	Orf1	CGCAGACGTGGCTGAGTACGT	PCR	140
2	Orf2	TGAGCTGGTTGGCGCTGTCCT		
3	ORFV-A32f-For	CTCCATTTAGAGGCCGTGAG	PCR/Sequencing	1098
4	ORFV-A32f-Rev	CGTGTTATGTGCCATCTTGC		
5	ORFV-A32i-For	GGTCGAGACAGCGCTTGA	Sequencing	-
6	ORFV-A32i-Rev	CGTCTACAACGCCGCCTAC		
7	ORFV-B2Lf-For	GACCTTCCGCGCTTTAATTT	PCR/Sequencing	1210
8	ORFV-B2Lf-Rev	CCCGCCTGCTAAAAGACT		
9	ORFV-B2Li-For	GTCCGCGTTCTTCCACTC	Sequencing	-
10	ORFV-B2Li-Rev	GCGGGCGTCAACTACTACA		
11	SP6	ATTTAGGTGACACTATAG	Sequencing	-
12	T7	TAATACGACTCACTATAGGG		

For the molecular characterization, DNA extracts from tissue homogenates were used. Fragments containing the full B2L and A32L genes were amplified by PCR in a reaction volume of 25 μL containing 500 nM forward primer, 500 nM reverse primer, 2 mM dNTPs, 1x PCR Buffer (Qiagen), 2.5 U Taq Polymerase (Qiagen) and 5 μL template DNA. The cycling conditions for the full A32L gene were: initial denaturation at 95 °C for 5 min, followed by 35 cycles at 95 °C for 50 s, 55 °C for 60 s and 72 °C for 90 s, and final extension at 72 °C for 7 min. The same protocol was used for the B2L gene except that the annealing was at 52 °C instead of 55 °C. For each PCR run, a negative control consisting of 5 μL of water instead of the template DNA was included. Aliquots of the PCR products were checked using electrophoresis on a 1.5 % agarose gel stained with ethidium bromide for 1 h at 100 V.

The amplified products of A32L and B2L genes were purified using Wizard SV Gel and PCR clean-up system kit (Promega), ligated into the pGEM-T vector (Promega) and transformed into SURE® 2 Supercompetent cells (Stratagene). Positive clones were selected and plasmids were purified using PureYield™ Plasmid Midiprep System (Promega). Sequencing of the plasmids was achieved using six primers indicated in Table 1. The plasmids were sequenced by LGC Genomics (Germany).

### Sequence and phylogenetic analysis

The sequences were assembled and edited using Vector NTI Advance™ 11.5 software (Invitrogen, Carlsbad, CA, USA). The full-length A32L and B2L gene sequences of the Ethiopian ORFV isolates (Table 2) together with those of homologue genes of parapoxviruses retrieved from GenBank (Table 3) were aligned using BioEdit software. All sequences were deposited in GenBank under accession numbers KT438513 to KT438530 for the B2L gene and KT438531 to KT438548 for the A32L gene.

**Table 2:** Ethiopian orf virus field isolates (*n* = 18) characterized in this study. The isolate’s names, area of collection, animal species and GenBank accession number for the A32L (ORF011) and B2L (ORF108) genes are provided

Sl No	Strain name	Area of collection	Animal species/Host	GenBank accession number	
				A32 gene	B2L gene
1	Adet/O01/2012	Adet	Sheep	KT438531	KT438513
2	Adet/O02/2012	Adet	Sheep	KT438532	KT438514
3	Adet/O03/2012	Adet	Sheep	KT438533	KT438515
4	Adet/O04/2012	Adet	Sheep	KT438534	KT438516
5	Adet/O05/2012	Adet	Sheep	KT438535	KT438517
6	Amba G/O01/2012	Amba Giorgis	Sheep	KT438537	KT438519
7	Amba G/C02/2012	Amba Giorgis	Goat	KT438536	KT438518
8	Amba G/O03/2012	Amba Giorgis	Sheep	KT438538	KT438520
9	ATARC/O01/2008	Adami Tulu	Sheep	KT438539	KT438521
10	ATARC/O02/2008	Adami Tulu	Sheep	KT438541	KT438523
11	ATARC/O01/2010	Adami Tulu	Sheep	KT438540	KT438522
12	ATARC/O02/2010	Adami Tulu	Sheep	KT438542	KT438524
13	Debre zeit/O01/2012	Debre zeit	Sheep	KT438543	KT438525
14	Debre zeit/O02/2012	Debre zeit	Sheep	KT438544	KT438526
15	Gondar z/C01/2013	Gondar zuria	Goat	KT438545	KT438527
16	Gondar z/C02/2013	Gondar zuria	Goat	KT438546	KT438528
17	Gondar z/C03/2013	Gondar zuria	Goat	KT438547	KT438529
18	Gondar z/O04/2013	Gondar zuria	Sheep	KT438548	KT438530

*ATARC* Adami Tulu Agriculture Research Center, *C* Caprine, *O* Ovine

**Table 3:** List of representative Parapoxvirus A32L (*n* = 12) and B2L (*n* = 13) genes nucleotide sequences retrieved from the GenBank for comparative sequence analysis and phylogenetic tree construction

Sl No	Strain	Gene name	Animal species	Country	GenBank accession no	Reference
1	ORFV-Nantou	A32L	Goat	Taiwan	EU327509	[17]
2	ORFV-NZ2	A32L	Sheep	New Zealand	DQ184476	[8]
3	ORFV-OV-IA82	A32L	Sheep	USA	AY386263	[1]
4	ORFV-OV-SA00	A32L	Goat	USA	AY386264	[1]
5	ORFV-67/04	A32L	Sheep	India	JN183066	[9]
6	ORFV-TN/2008	A32L	Sheep	India	JN183076	[9]
7	ORFV-Mys/2010	A32L	Sheep	India	JN183075	[9]
8	ORFV-Meg/2003	A32L	Goat	India	JN183073	[9]
9	ORFV-59/05	A32L	Goat	India	JN183074	[9]
10	ORFV-Ass/2010	A32L	Goat	India	JN183069	[9]
11	PCPV-VR634	A32L	Bovine	New Zealand	GQ329670	[18]
12	BPSV-BV-AR02	A32L	Bovine	USA	AY386265	[1]
13	ORFV/2009	B2L	Goat	Korea	GQ328006	[19]
14	ORFV/2003	B2L	Goat	USA	AY278208	[20]
15	ORFV/2004	B2L	Sheep	USA	AY424970	[21]
16	ORFV-India 82/04	B2L	Goat	India	DQ263303	[22]
17	ORFV-India 67/04	B2L	Sheep	India	DQ263305	[22]
18	ORFV-India 79/04	B2L	Sheep	India	DQ263306	[22]
19	ORFV/GanSu/2009	B2L	Sheep	China	HQ694772	Unpublished
20	ORFV-Assam/09	B2L	Goat	India	JN846834	[23]
21	ORF-Vaccine strain	B2L	NA	USA	AY278209	[20]
22	ORFV-D	B2L	Sheep	Brazil	JN088052	Unpublished
23	ORFV-NE2	B2L	Goat	Brazil	JN088051	Unpublished
24	PCPV	B2L	Sheep	USA	AY424972	[21]
25	BPSV	B2L	Sheep	USA	AY424973	[21]

For phylogenetic reconstructions of the A32L and the B2L gene trees, sequences were aligned with Muscle using the aligned codon option of MEGA 6 [24]. The file with aligned sequences was exported into MrBayes compatible Nexus file using Mesquite 2.74. For both A32L and B2L genes, the Tamura 3-parameter model of nucleotide substitution with gamma-distributed rate variation across sites was selected as best-fit model with MEGA 6. MrBayes3.2.2 was then used to produce a tree.

The MCMC method was run for 10,000,000 generations, with a sampling frequency of 1,000, until model convergence was reached. Four independent chains were run with the chain heating temperature set to 0.2. At the convergence, 25 % of the initial trees were discarded for all analyses. A 50 % majority rule consensus tree was built from the resulting trees. The tree was visualized using Figtree 1.4.2 program. Bovine popular stomatitis virus and pseudocowpox virus were used as out-groups.

Additionally, the amino acid sequences of the ATPase were aligned, using BioEdit 7.2.3, to analyze the presence of five predicted functional motifs and its C-terminal heterogeneity. For a more comprehensive analysis of the C-terminal heterogeneity, all publicly available aa sequences of ORFV ATPase protein were included.

## Results

### Outbreaks description, preliminary screening and viral isolation

**Fig. 2:**
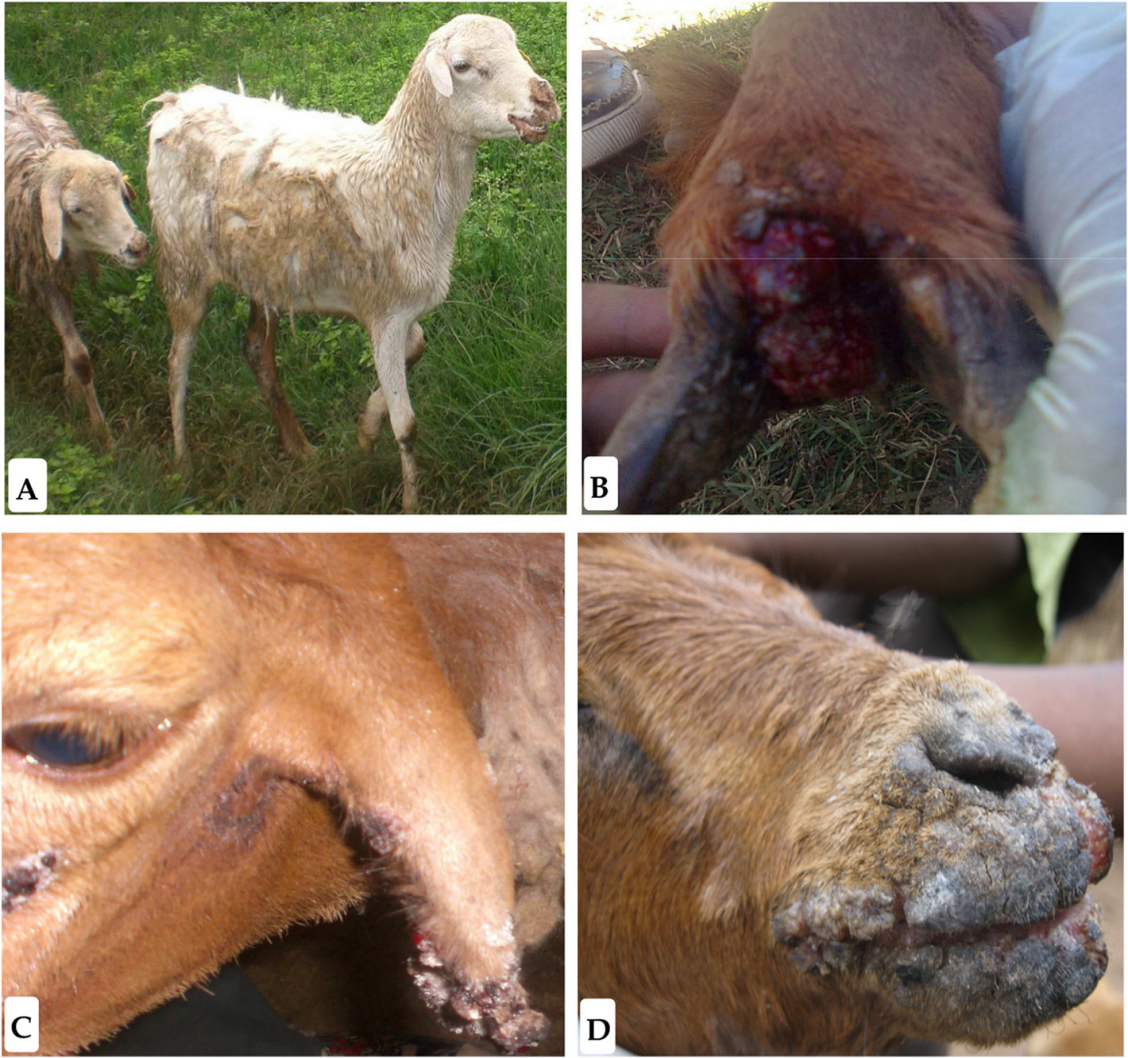
Representative clinical sign of orf virus infection observed during outbreaks investigation. **a** Scabby and ulcerated ecthyma lesions around oral commissures, muzzle, lower jaw and knee region in sheep. **b** Severe bloody tissue growth lesion in the interdigital space in sheep. **c** Growth of tumor-like lesions on the ear skin in sheep. **d** Severely crusted wart-like multiple lesions on the oral commissures, lips and nostril area in goat

The first orf outbreak investigated in this study occurred in November 2008, in sheep reared at the Adami tulu agriculture research center (ATARC) causing specific lesions (Fig. 2a) in 10 % of the animals. The outbreak occurred 4 months after vaccination of the animals with a capripox vaccine, strain KS1-O180, leading the researchers of this center to suspect capripoxvirus infections. In 2010, the same center experienced a second outbreak of pox disease in sheep with a morbidity rate of about 3 %. The third outbreak occurred in June 2012 at the Adet sheep research center where the diseased sheep presented with pox lesions at different sites of the body (Fig. 2b) with a morbidity rate of 22 % (25/112). During the same month, on 26 June 2012, the fourth outbreak occurred in Debre Zeit, where an animal owner presented two diseased sheep to the veterinary clinic with lesions around the muzzle and nostrils (Fig. 2c and d).

The fifth and the sixth outbreaks were in the Amba Giorgis on 21 November 2012 and, in Gondar zuria on 20 January 2013 in sheep and goats reared by individual farmers sharing common grazing land and pasture. Animals presented with lesions on the muzzle, nostril and interdigital spaces and the morbidity rates were estimated to be around 4 % and 3 %, respectively.

In general, for all outbreaks areas, there was no recorded mortality associated with ORFV infections and no human infections were reported. Animals which had developed severe lesions around the mouth and interdigital spaces were unable to walk and graze freely which resulted in a reduction in body weight and production loss. The dry crusted skin lesions led to secondary complications and worsened the health of the animal. Severe lesions were observed in young lambs and kids which had close contact with diseased parents. Eighteen out of 24 samples collected during these outbreaks tested positive using ORFV specific PCR primers orf 1 and orf 2 which produced the expected fragment size of 140 bp (Table 1). The distribution of the positive samples was as follows: ATARC, 2008 (2/4), ATARC, 2010 (2/2); Adet 2012 (5/6); Debre zeit 2012 (2/2); Amba Giorgis 2012 (3/4); Gondar zuria 2013 (4/7). For each positive sample, the complete B2L and A32L genes were amplified, sequenced and used for comparative analysis.

>Infected Vero cell cultures developed characteristic poxvirus-induced cytopathic effects (CPE), with rounded cell detaching from monolayer, after 3–5 days in culture for either the first or second blind passage (data not shown). This result, taken together with the results from PCR screening, confirmed the presence of infectious ORFV in all attended herds.

### Sequence analysis and phylogenetic tree construction

Comparative sequence analysis of the Ethiopian ORFV isolates based on A32L and B2L genes was carried out including homologous genes from a selection of parapoxviruses (Table 3) using MEGA.6. The arithmetic means of nucleotide and amino acid substitutions per site between the Ethiopian isolates only (within group mean distance) and between the Ethiopian isolates and foreign isolates (between groups mean distance) were estimated using MEGA. The within group mean distance was 0.01 at both the nucleotide and aa levels for the Ethiopian isolates based on their A32L gene nucleotide sequences and the deduced aa sequences. The mean distance between Ethiopian isolates and foreign isolates was 0.020 at both the nucleotide and aa levels. Likewise, for the B2L gene, the within group mean distance for the Ethiopian isolates was 0.010 at both the nucleotide and aa levels. The mean distance between Ethiopian isolates and foreign isolates was 0.020 at both the nucleotide and aa levels. This shows that the Ethiopian ORFV isolates are more similar to each other than to foreign isolates.

**Fig. 3:**
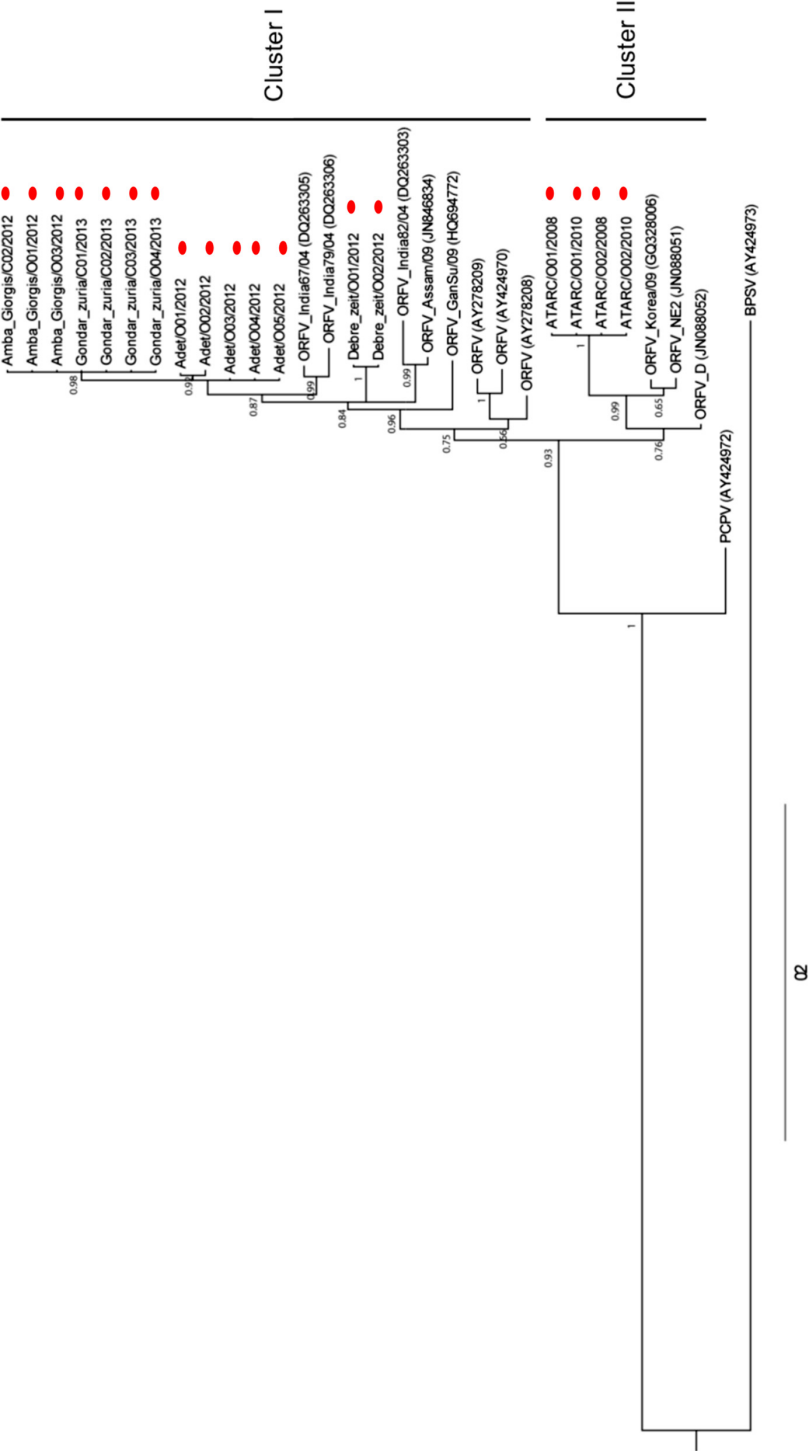
Phylogenetic tree of the B2L gene nucleotide sequences (1137 nt) of Ethiopian orf virus isolates. Eighteen Ethiopian orf virus isolates and eleven foreign orf viruses were included. In addition, the homologue gene sequence of BPSV (AY424973) and PCPV (AY424972) and were used as out-groups. The numbers displayed on the branches represent the posterior probabilities. The sequences retrieved from GenBank are indicated with accession number under bracket and those of this study were highlighted with red bubbles

In the phylogenetic tree reconstructions, based on the nucleotide sequences of the B2L gene, the Ethiopian ORFV isolates grouped into two major clusters. Cluster I included fourteen local ORFV isolates (Adet, Amba Giorgis, Gondar zuria and Debre zeit) and eight foreign ORFV strains from India, Taiwan and USA; whereas the four ATARC ORFV isolates and three strains from New Zealand, Taiwan and USA were grouped within cluster II (Fig. 3).

**Fig. 4:**
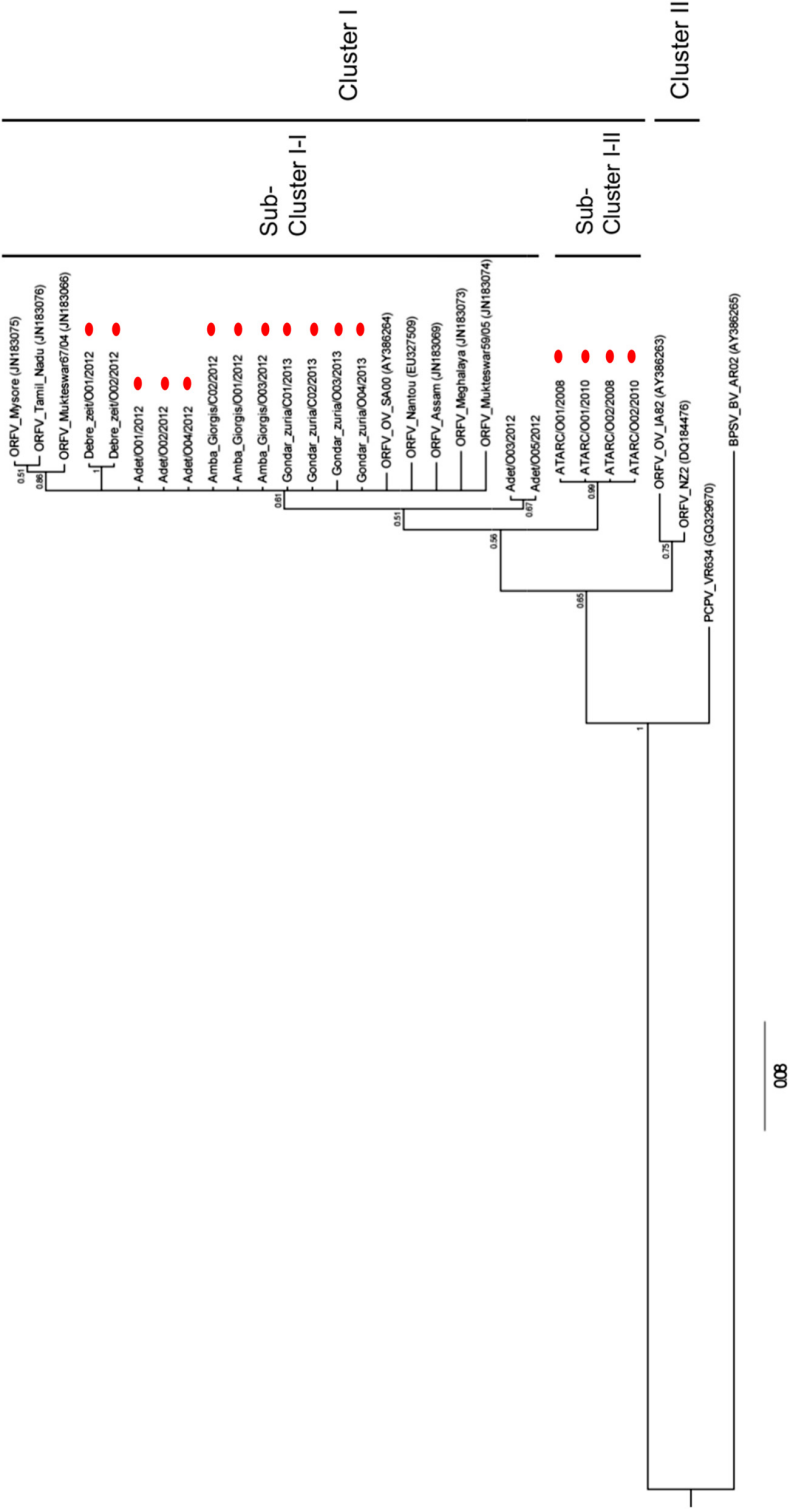
Phylogenetic tree of the A32L gene nucleotide sequence (825 nt) of Ethiopian isolates orf virus isolates. Eighteen Ethiopian orf virus isolates and ten foreign orf viruses were compared. Additionally, the homologue gene sequence of BPSV (AY386265) and PCPV (GQ329670) were used as out-groups. The numbers displayed on the branches represent the posterior probabilities. The sequences retrieved from GenBank are indicated with accession number under bracket and those of this study were highlighted with red bubbles

In the A32L gene tree, two major clusters were observed, with all Ethiopia isolates falling within cluster I. However, the four ATARC isolates formed a separate sub-cluster as compared to the other Ethiopian and foreign ORFV isolates of this cluster I (Fig. 4).

### C-terminal heterogeneity of Ethiopian ORFVs ATPase

The analysis of the aa sequences of Ethiopian ORFV isolates confirmed the high level of conservation of the N-terminal region of the ATPase encoded by the A32L gene. No aa change was observed until residue position 249. All predicted five functional motifs of the ATPase protein were found in all Ethiopian ORFV isolates sequences.

In contrast to the N-terminal, the C-terminal displayed a high variability among the Ethiopian isolates and also when compared to foreign isolates.

**Fig. 5:**
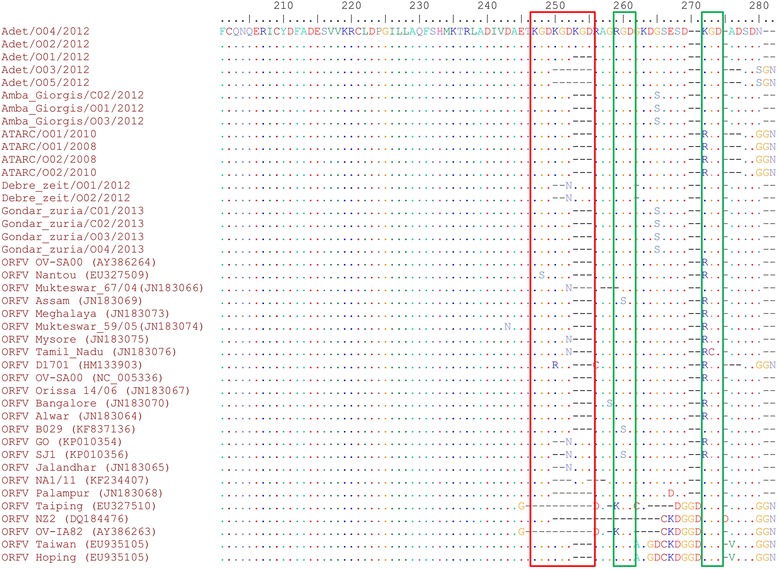
Multiple sequence alignment of the deduced amino acid sequence of the A32L gene of the Ethiopian orf virus isolates. The deduced amino acid sequence of the A32L gene of twenty four foreign orf virus strains retrieved from GenBank were added of comparison. The analysis revealed a high divergence in the carboxyl terminal region of ATPase protein sequence unlike the N-terminal region. The three repeats of KGD amino acid residues are highlighted with red color box. The two green color boxes indicate the presence of RGD sequences at two sites

Six different profiles were observed at the C-terminal of the ATPase protein of the Ethiopian ORFV isolates (Fig. 5). Two isolates, Adet/O02/2012 and Adet/O04/2012, contained repeats of three KGD sequences (position 247 – 255), a KGD at position 270–272, and their protein sequences ended with an ADSND. The ATPase protein of isolate Adet/O01/2012 had two repeats of KGD sequences (position 247 – 252), a KGD at position 270–272 and ended with an ADSND. The ATPase protein sequence of isolate Adet/O01/2012 was completely similar to that of the ORFV isolate Orissa 14/06 from India.

All three ORFV isolates from Amba Giorgis, and four from Gondar zuria had two repeats of KGD sequences (position 247 – 252), a KGD at position 270–272, and ended with an ADSND. Additionally, these isolates presented a G265S aa substitution when compared to other Ethiopian ORFVs and all publicly available ORFV ATPase protein sequences.

The four isolates from ATARC also presented two repeats of KGD sequences (position 247 – 252) at the C-terminal of the ATPase protein, but a RGD was present at position 270–272 instead of a KDG, and ended with a SDGGN.

The isolates Adet/03/2012 and Adet/05/2012 had only one KGD at position 247–249, a KGD at position 270–272 and their ATPase protein sequence ended with a SGSGN.

The two isolates from Debre zeit presented two KGDs, at position 247–249 and 253–255 interrupted by a two aa deletion at position 250–251 and an N at position 252, and their sequence ended with an ADSND. Additionally they presented a deletion of a G at position 262.

An additional copy of RGD sequence was present at position 259–261 of all sequenced isolates, but was not specific to the Ethiopian isolates.

## Discussion

ORFV infection of sheep and goats is generally neglected worldwide due to the low morbidity rates. In Ethiopia, despite the increased frequency of orf disease outbreaks, there hasn’t been any attempt to further investigate the disease.

This study describes for the first time, the molecular characterization of ORFV isolates collected from sheep and goats during six suspected orf disease outbreaks in Ethiopia.

Our phylogenetic analyses suggest two main clusters of ORFV infecting sheep and goats in Ethiopia. All isolates collected from both sheep and goats in Amba Giorgis, Gondar zuria, Adet and Debre zeit grouped in cluster I of the B2L tree, while those from ATARC were located separately in cluster II.

In addition to the clear separation of the Ethiopian ORFV isolates into 2 clusters on the B2L tree, two isolates from Debre zeit further diverged from other isolates of cluster I, forming their own sub-cluster.

The analysis of the C-terminal domain of their A32 protein revealed six different amino acid profiles among the Ethiopian ORFV isolates, of which the G265S was unique to the Ethiopian ORFV isolates of Amba Giorgis, Gondar zuria, and the SGSGN ending of the protein was present only in isolates Adet/03/2012 and Adet/05/2012 of Ethiopia. Additionally, two ORFV isolates from Debre zeit also showed a unique deletion of a G at position 265.

Overall the Ethiopian ORFV isolates showed high similarity to previously sequenced foreign ORFV isolates on either the B2L or the A32L gene. Nevertheless, they carried specific mutations that differentiated them from previously sequenced isolates [1, 4, 8–11].

Interestingly, there was a good agreement between the phylogenetic analysis and the profile of the C-terminal of the Ethiopian isolates. All ATARC isolates of the cluster II presented two RGD attachment sequences in the C-terminal of the ATPase gene at position 259–261 and 270–272, whereas other Ethiopian isolates belonging to cluster I contained only one RGD at position 259–261. The RGD at position 270–272 was replaced by a KGD for the Ethiopian ORFV isolates of cluster I.

We have also noticed the presence of variant KGD repeat motifs of one to three copies in all Ethiopian ORFV isolates. Although, these KGD motifs were found in previously sequenced ORFV isolates [1, 4, 8–11], the presence of a repeat with 3 consecutive KGD units was a unique feature of two Ethiopian isolates (Adet/O02/2012 and Adet/O04/2012) collected in Adet research centre. Interestingly, other isolates collected from the same outbreak, presented with either 2 KGD repeats (isolate Adet/O01/2012) or only 1 KGD (Adet/03/2012 and Adet/05/2012).

The presence of all three KGD repeat variants within the same farm highlights the possibility of a rapid mutation of the A32L gene, probably during chronicity, or during adaptation to new tissues or to improve virus shedding and increase its spreading potential.

It is interesting to note that the Adet research centre, where all 3 variants of KGD motifs were recorded, also presented the highest morbidity rate as compared to other outbreak areas indicating a high infectivity rate. However, the fact that the animals of the Adet research centre are reared in a confined environment could also increase the chance of ORFV transmission from infected animals to those susceptible.

In addition to the major clinical signs of orf disease in sheep and goats, as described elsewhere [25–27], we have also documented the appearance of less specific skin lesions in areas other than the nose and mouth which could be easily confused with sheep pox, goat pox, peste des petits ruminants, FMD and dermathophilosis lesions [3]. This could explain why the ATARC researchers suspected capripox disease during the first outbreak that occurred on their farm, four months after vaccination against sheep pox. This highlights the need to perform laboratory confirmation of ORFV suspected infection using molecular tools.

Since ORFV infections may induce lesions that are similar to other economically important diseases, it is urgent to adopt multiparamatric pathogen detection methods for screening samples from animals presenting pox-like lesions. The screening for eventual asymptomatic carriers as well as wildlife is also needed to identify potential reservoirs.

An important reason for investigating orf disease remains it zoonotic potential [3]. Although no human infections were reported in this study, it is not possible to completely rule out the presence of zoonotic ORFV infections in Ethiopia, as human infections may be self-limiting, and occur without the presence of severe disease [28].

## Conclusion

This paper reports the first characterization of ORFVs circulating in Ethiopia. It shows that two main clusters of ORFVs were involved in the investigated outbreaks in Ethiopia.

The endemicity of orf in the country calls for the urgent adoption of appropriate diagnostic tools and a better monitoring of the isolates circulating within the country.

The use of appropriate personal protective equipment such as gloves and proper hygiene while handling diseased animals and carcasses of dead animals is also recommended to reduce the risk of zoonotic infections.

## Additional file

Additional file 1:
**Table S1.** Questionnaire format for data collection during orf suspected outbreak investigations. (PDF 66 kb)
