# Supplementary material for: Molecular characterization of orf virus from sheep and goats in Ethiopia, 2008–2013
Source: Virol J. 2016 Feb 29;13:34. doi: 10.1186/s12985-016-0489-3 (PMC4770539; doi:10.1186/s12985-016-0489-3)
Supplement: Additional file 1: — Table S1. Questionnaire format for data collection during orf suspected outbreak investigations. (PDF 66 kb) [file 12985_2016_489_MOESM1_ESM.pdf]

**Questionnaire format for orf disease outbreaks investigations**

1. Background information:

    Today's date________________________________

    Owner's name________________________________

    Region/Zone/District________________________________

    Date of first outbreak _____________________

    Type of farming   Free range ☐   Penned ☐

2. Do you have only sheep ☐  only goats ☐  or both ☐

3. Number of sheep and goats per household____________

4. Occurrence of pox-like infections:

    4.1. Occurrence

    First time   Yes ☐   No ☐

    Commonly occurred   Yes ☐   No ☐

    4.2. Season of occurrence

    Dry season   Yes ☐   No ☐

    Rainy season   Yes ☐   No ☐

5. Animals affected/sick (sheep/goat/both):

| Species | sex | Age group | | | Total |
|---|---|---|---|---|---|
| | | <1 years | 1-3 years | >3 years | |
| | Male | | | | |
| | Female | | | | |

6. Major clinical signs observed: __________________________________________________

7. Animals dead:

| Species | sex | Age group | | | Total |
|---|---|---|---|---|---|
| | | <1 years | 1-3 years | >3 years | |
| | Male | | | | |
| | Female | | | | |

8. Possible source of the outbreak:

Introduction of infected animals Yes ☐ No ☐

Contact at communal grazing/watering points Yes ☐ No ☐

Contact at market places Yes ☐ No ☐

9. Vaccination status:

Does sheep/goat vaccinated? Yes ☐ No ☐

If yes, for which disease? ___________________

When the vaccination performed? ___________________

10. Does the animal get treatment? _______________________

11. Was any skin lesions observed in animal handlers? Yes ☐ No ☐

If yes, in which part of the body? ___________________

12. Sample collection information

| Serial Number | Sample identification code | Animal species | Type of sample collected | Collection site in the Body | Sample collection date |
|---|---|---|---|---|---|
| | | | | | |
| | | | | | |
| | | | | | |
| | | | | | |
| | | | | | |
| | | | | | |
| | | | | | |
| | | | | | |
| | | | | | |
